# Optimization of *Awaze* paste formulations: The effects of using spices through a mixture design approach

**DOI:** 10.1016/j.heliyon.2024.e35141

**Published:** 2024-07-24

**Authors:** Biadge Kefale, Mulugeta Admasu Delele, Solomon Workneh Fanta, Solomon Abate

**Affiliations:** a Faculty of Chemical and Food Engineering, Bahir Dar Institute of Technology, Bahir Dar University, Ethiopia; b Ethiopian Institute of Agricultural Research, Holeta Agricultural Research Centre, Food Science and Nutrition Research, Holeta, Ethiopia; c Ethiopian Institute of Agricultural Research, Head Quarter, Food Science and Nutrition Research, Addis Ababa, Ethiopia

**Keywords:** Formulation, Ingredients, *Awaze* paste, Nutritional quality, Microbial safety

## Abstract

Previous studies have revealed the microbial quality of *Awaze* paste. However, limited reports describe the effect of individual spices on *Awaze* paste quality. A mixture design approach was used to determine the appropriate proportions, with 15 experimental points for independent variables including RP (60–90 %), GA (10–30 %), RO (5–20 %), and GI (5–10 %). The techno-functional properties, particle size, antioxidant activity (DDPH radical assay), proximate composition, iron (Fe), zinc (Zn) content, viscosity, hardness, and microbiological quality of *Awaze* paste were assessed. The prepared *Awaze* paste showed a range of characteristics, with antioxidant activity (DDPH radical assay) ranging from 11.86 % to 62.5 %, crude protein content from 6.18 % to 16.22 %, crude fat from 5.7 % to 12.6 %, crude fiber from 16.86 % to 29.06 %, total ash content from 6.32 % to 9.94 %, total carbohydrate from 41.79 % to 60.61 %, energy from 264.3 to 329.2 k cal. , iron (Fe) content from 35.59 to 108.82 mg/100g, zinc (Zn) content from 1.72 to 26.93 mg/100g, viscosity from 65.5 to 125.5 cps, hardness from 8.48 to 55.09 g, yeast and mold count from 0.83 to 2.04 log cfu/g, and total bacterial count from 1.53 to 2.61 log cfu/g. Significant differences (p < 0.05) were observed in proximate composition, techno-functional properties, particle size, antioxidant activity, physicochemical properties, and microbiological characteristics among the formulations of *Awaze* paste. The selected formula showed a statistically significant difference (p < 0.05) compared to the control sample. The formulation containing 74.79 % RP, 10 % GA, 10.2 % RO, and 5.0 % GI was determined to be the optimal formula with a desirability of 0.73, based on the evaluated parameters. This preferred *Awaze* paste had a porosity of 28.12 %, particle size of 16.49 μm, antioxidant activity of 63.63 %, crude protein content of 17.28 %, iron (Fe) content of 98.06 mg/100g, and zinc (Zn) content of 15.04 mg/100g. Therefore, this optimal blend of ingredients could be used to produce a consumer accepted *Awaze* paste.

## Highlights

• Increasing the proportion of RP and GI in the formulation significantly enhances the iron (Fe) and zinc (Zn) content, while GA improves the flow ability, antioxidant activity, and particle size of *Awaze* paste.
• Total bacteria, yeast, and mold counts were found to be within safe levels for formulations according to international food safety standards.
• Optimization of the ingredient mixing ratio suggests a formulation of 74.79 % RP, 10 % GA, 10.2 % RO, and 5.0 % GI.

## Graphical abstract

**Figure:**
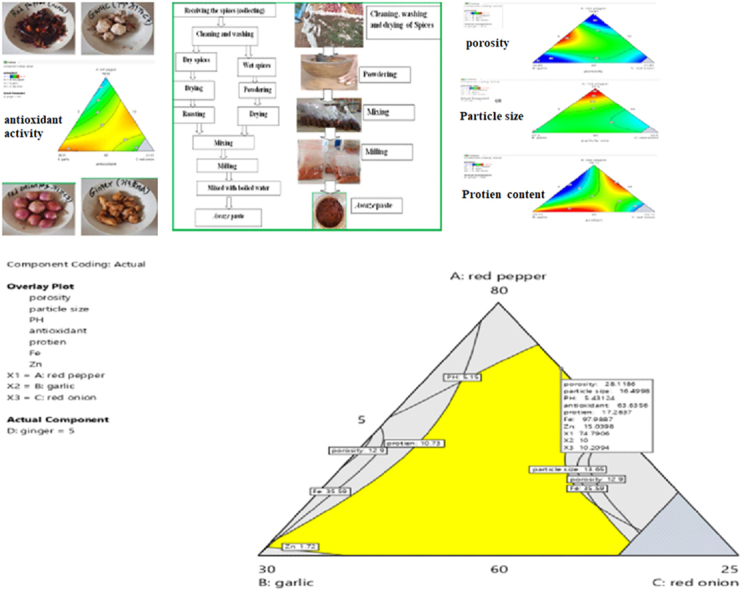

## 1 Introduction

Food formulation is important for developing consumer preferred new food products that offer health and nutritional benefits. Food formulation is the process of developing food products with the goal of meeting nutritional and dietary needs [1]. Micro-nutrient deficiencies such as zinc (Zn), iron (Fe), and vitamin A pose a significant world health problem. The majority of malnourished individuals worldwide, over 80 %, are found in developing countries [2]. Food formulation using staple foods like wheat, maize, and rice in sub-Saharan Africa has been identified as a strategy to address both macro-nutrient and micro-nutrient deficiencies [3]. In this region, various approaches are employed to tackle malnutrition, including formulating complementary foods from ingredients like rice, faba bean, peanut oil, and sweet potato, which are rich in vitamin A and iron (Fe) content. In Ethiopia, significant efforts have been made to combat malnutrition [4]. The prevalence of chronic malnutrition (stunting) has decreased from 58 % to 38.4 % and wasting decreased from 12 % to 9.9 % between 2000 and 2016. This decrease is due to the use of nutritionally enhanced agricultural products, food formulation, and fortification [5].

Fermented *Awaze* paste is a spicy Ethiopian traditional cuisine used to make stew (*Wot*) in rural households and as a seasoning for spicy food. It is prepared mainly from RP, GI, GA, and RO with small proportions of fenugreek, basil, thyme, rosemary, *mekelesha*, cinnamon, white cumin, black cumin, and rue. These ingredients contain high amounts of antioxidants, minerals, and fiber, including carotenoids.These compounds, with expressed antioxidant and anti-inflammatory activities that improve scar formation, decrease blood cholesterol levels, and improve stamina [[6], [7], [8]]. The amount and characteristics of flavor, color, and especially pungent behaviour of red pepper are important quality parameters. The pungency, aroma, and availability in different colors, shapes, and sizes are important characteristics of red pepper that make it favourable for use as an ingredient in the production of several foods [[9], [10], [11], [12]]. Garlic is a common food spice used widely for its medicinal value, as a preservative, and for its flavor quality [[13], [14], [15]].

Among the main ingredients, red onion has a significant contribution in terms of nutritional value to the human diet as well as improving the taste, flavor, and aroma of food [[16], [17], [18]]. Ginger is also important in the production of oleoresin and oil due to its high content of bio-active compounds [[19], [20], [21]]. Spices are well-known food ingredients that enhance the flavor and aroma of foods. Nutritionally, spices play a significant role in reducing fatty acid oxidation and preserving foods, and they also have a vital role in food formulation due to their significant nutritional and sensory quality, as well as their health benefits [22,23].

Previous studies have examined the physicochemical and microbiological quality of laboratory-made *Awaze* paste. The microbiological quality of *Awaze* paste during fermentation at different time intervals was reported [24]. The behaviour of *E. coli* under different storage conditions was studied [25]. The role of probiotics in lactic acid bacteria isolated from *Awaze* paste were studied *in vitro* [26,27].

Mixture design is a crucial tool in the design of formulation of food products, especially for foods prepared with many ingredients, to study the influence of each individual ingredient on the food characteristics. This helps in finding the optimum formula based on the desired quality of the product [28,29]. Optimal mixture design is a useful tool for determining the optimum formula by considering the best quality characteristics of foods [29,30].

There is limited research in the literature on the formulation of *Awaze* paste. Women in Ethiopia prepare *Awaze* paste at the household level, but the proportion of RP, GI, RO, and GI varies from women to women. There is no standard method of preparation for *Awaze* paste, which affects the characteristics of the final food product due to variations in ingredient proportions. Therefore, the objective of this work was 1) to formulate a composite powder of spices from RP, GA, RO, and GI for an acceptable *Awaze* paste and 2) to evaluate the techno-functional properties, particle size, antioxidant activity, proximate composition, iron (Fe) content, zinc (Zn) content, viscosity, hardness, and microbiological properties of enriched *Awaze* paste.

## 2 Materials and methods

### 2.1 Materials

The experiment was done in October 2022 at the Holeta Agricultural Research Centre (HARC) in Ethiopia. A sample of RP was obtained from the Bure district in the Amhara region of Ethiopia, due to its potential for red pepper production [31]. Additionally, GA, RO, and GI were collected from HARC. The spice ingredients needed in small amounts were collected from the Holeta market in October 2022 in the Oromia region of Ethiopia. These spices were fenugreek, cardamom, black cumin, cinnamon, ground pepper, clove, rue, *kemune, gewze*, thyme, coriander, white cumin, basil, long pepper, and rosemary. After collecting all the required ingredients, *Awaze* paste was prepared and techno-functional properties, proximate composition, iron (Fe) content, zinc (Zn) content, viscosity, hardness, and microbiological quality were determined. Analytical grade chemicals and reagents were used for this work.

### 2.2 Experimental design for mixing ratio optimization

The main goal of the experimental design was to maximize the selected dependent variables that serve as the best quality criteria for *Awaze* paste. These variables include porosity, particle size, antioxidant activity, crude protein content, as well as zinc (Zn) content and iron (Fe) content through optimization of dependent variables. In the experiment, the ratio of ingredients was set according to the household preparation protocol commonly used by many women in the districts where *Awaze* paste could potentially be utilized. Four independent variables, including RO, GA, RO, and GI, were considered in the experimental design for their proportions. The minimum and maximum ranges for all four parameters are provided to create an experimental design. Based on the mixture design, a total of 15 formulations were obtained. The study included constraints of RP (X1: 60–90 %), GA(X2: 10–30 %), RO (X3: 5–20 %), and GI(X5: 5–10 %).

Numerical and graphical optimization were used to find the best ingredient mixing ratio and predict experimental points for the mixture.The ingredients RO was set to the maximum, while RP, GA, and GI were set to the range for the numerical optimization. Crude protein content, antioxidant activity, zinc (Zn), and iron (Fe) content were set to maximum, while the other parameters were set to in the range. Crude protein content, antioxidant activity, zinc (Zn), and iron (Fe) content were given high relative importance of “5’’ in the work sheet. A significant test was conducted using an F-test for the model for analysis. The effects of changing each mixture constituent were visualized through contour plots of the selected model. The formulated *Awaze* paste was compared to a control sample based on selected parameters. The crude protein content, antioxidant activity, zinc (Zn), and iron (Fe) content of the *Awaze* paste product were evaluated.

All the formulations were as follows: F stands for formulation, RP stands for red pepper, GA stands for garlic, RO stands for red onion, and GI stands for ginger:

F1: 60% RP + 20.22 % GA + 12.01% RO + 7.77% GI

F2: 67.28% RP+ 19.37 % GA + 8.28% RO+ 5.07% GI

F3: 70.13% RP+ 10 % GA + 9.87% RO + 10% GI

F4: 68.84% RP + 13.99% GA + 12.16% RO + 5.01% GI

F5: 60% RP + 30 % GA + 5% RO + 5% GI

F6: 76.39% RP + 10 % GA+ 8.6% RO + 5.01% GI

F7: 64.55% RP + 15.46 % GA + 10.17% RO + 9.82% GI

F8: 60% RP + 15% GA + 20% RO + 5% GI

F9: 68.88% RP + 16.1% GA + 5.02% RO + 10% GI

F10: 65.27% RP + 10% GA + 17.7% RO + 7.03% GI

F11: 74.39% RP + 13.33% GA+ 5% RO+ 7.28% GI

F12: 63.27% RP + 24.68% GA + 7.03% RO + 5.02% GI

F13: 60% RP + 10.13% GA+ 19.87% RO + 10% GI

F14: 64.15% RP + 20.85% GA + 5% RO + 10% GI

F15: 60% RP + 14.81% GA + 15.2% RO + 9.99% GI

### 2.3 Control sample

The control sample collection area was chosen based on the availability of the product in the area, as well as the frequency of production and utilization of *Awaze* paste. It was obtained from bure district of Amhara region. It was prepared at the household level using ingredients identical to those used for the formulated *Awaze* paste. However, the ratio of the ingredients used in the control sample differed from formulated *Awaze* paste.

### 2.4 Product development of *Awaze* paste

#### 2.4.1 Preparation of ingredients

*Awaze* paste was prepared following traditional household methods as described [24]. Dry and wet spices were involved in the *Awaze* paste product development. Dry spices such as fenugreek, cardamom, red pepper, long pepper, cinnamon, clove, *kemune*, coriander, black cumin, white cumin, and ground pepper were combined with wet spices like ginger, basil, red onion, garlic, rue, thyme, and rosemary to prepare the paste. The dried and cleaned RP was pulverized with a wooden mortar and pestle.Wet spices, including GA, RO, and GI, were washed and peeled. After cleaning, washing, peeling, sun-drying, and lightly roasting of both dry and wet spices, each formulation was done as follows.

The independent variables (RP, GA, RO, and GI) were mixed based on the formula obtained from mixture design. Controlled variables consisted of 5 g of fenugreek, 5 g of cardamom, 2.5 g of basil, 2.5 g of white cumin, 1.25 g of *mekelesha*, 1.25 g of black cumin, 1 g of rue, 0.5 g of coriander, 0.4 g of thyme, 0.4 g of rosemary, and 20 g of salt, which were constantly added to each formulation. The mixed spices were milled using a laboratory mill (Perten Instruments, Finland). After milling the mixed spices, 200 g of composite powder was added to 300 mL of boiled water and mixed until it became thick consistency. A control sample was used for comparison with the formulations.

### 2.5 Proximate composition

The moisture content, crude protein, crude fiber, crude fat, total ash, total carbohydrate and energy value of each formulation were determined using the official method [32].

### 2.6 Techno- functional property of the composite powder

#### 2.6.1 Water absorption capacity(WAC)

The WAC was determined following the method [32]. A 1 g of the sample was mixed with 10 mL of distilled water in a centrifuge tube. The solution was allowed to stand at 30 °C for 1 h, then centrifuged at 2000 rpm for 30 min. The supernatant volume of the composite powder was then measured in a 10 mL measuring cylinder. The WAC was calculated following the equation provided below (1):

(1) WAC=10−Volumeofwaterleftunabsorbed

#### 2.6.2 Bulk density (BD)

The BD of the formulations was analyzed following the method described [33]. For each formulation, 50 g of the composite powder of *Awaze* paste was added to a 100 mL measuring cylinder, and the volume was then measured. The BD was determined following the equation provided below (2):

(2) $$\text{BD}=\frac{\text{Weight}\;\text{of}\;\text{powder}\;\text{sample}}{\text{Volume}\;\text{of}\;\text{powder}\;\text{sample}}$$

#### 2.6.3 Tapped density (TD)

The TD of the formulations was determined following the method [33]. The TD of each formulation was determined by adding 50 g of sample to a measuring cylinder and manually tapping the cylinder vertically until the height of the powder in the cylinder did not change. The TD of *Awaze* paste was calculated according to the equation provided below (3):

(3) $$\text{TD}=\frac{\text{Mass}\;\text{of}\;\text{taping}}{\text{Volume}\;\text{of}\;\text{taping}}$$

#### 2.6.4 Porosity (*ε*)

The porosity of the formulation was determined using the formula *ε* (%) [34]. The porosity of *Awaze* paste was determined according to the equation provided below (4):

(4) $$ɛ\;\left(\%\right)=\left[1-\frac{\text{BD}}{\text{TD}}\;\right]\;x\;100$$

Where BD = bulk density and TD = tapped density.

#### 2.6.5 Hausenr ratio (Hr) and Carr's index (CI)

The HR and CI of the samples were calculated using the following formula [34]. The HR and CI of *Awaze* paste were determined according to the equation provided below (5 and 6):

(5) $$\text{Hr}=\frac{\text{TD}}{\text{BD}}$$

(6) CI=(TD−BD)x100/TD

Where BD = bulk density and TD = tapped density.

### 2.7 Particle size

The particle size of the formulated *Awaze* paste was determined using a laser diffraction particle size analyzer (Master sizer 2000; Malvern Instruments Ltd., Worcestershire, UK) following the method [35].The composite powder of the formulated *Awaze* paste samples were dispersed in water (ISO 13320-1) using the master sizer instrument. In the particle size distribution curve of the analyzed sample, D_10_, D_50_, and D_90_ representing 10 %, 50 %, and 90 % were used to describe the size of particles and D (4, 3) and the specific surface area (m^2^/g) were used to express volume-weighted mean for each formulations.

### 2.8 Physicochemical property of *Awaze* paste

#### 2.8.1 pH value

The pH value of the prepared *Awaze* paste was obtained according to the method [36]. A 10 g *Awaze* paste sample was mixed with 100 mL of distilled water, and the pH value was determined using a calibrated pH meter (Mettler Toledo, China).

#### 2.8.2 Titratable acidity (TA)

The TA of *Awaze* paste was determined following the method [36]. A 10 g of prepared *Awaze* paste were dissolved in 100 mL of distilled water. The mixture was stirred gently and allows to stand for 1 h. A 10 mL of the solution were then taken, and 0.5 mL of phenolphthalein was added.The solution was titrated with 0.1 M NaOH until a pink color appeared. The TA of *Awaze* paste was determined according to the equation provided below (7):

(7) $$\text{TA}\;\left(\%\right)=\frac{\text{Vol}.\text{NoH}\;\text{used}\left(\text{mL}\right)x0.1\;M\;\text{NaoH}\;x\;\text{milli}\;\text{equvalant}\;\text{factorx}100}{\text{mass}\;\text{of}\;\text{sample}\left(g\right)}$$

#### 2.8.3 Total soluble solids

The TSS of *Awaze* paste were obtained following the method [32]. A 10 g of *Awaze* paste were dissolved in 50 mL of distilled water. The mixture was then filtered, and a 1 mL aliquot was placed in a digital refractometer. The result was recorded as ^o^ Brix.

#### 2.8.4 Antioxidant activity

The antioxidant activity of the formulation and control samples was determined following the method [37]. For each formulated *Awaze* paste, a 400 mg *Awaze* paste was placed in a 15 mL centrifuge tube with 5 mL of a solvent mixture MeOH: H_2_O (80:20, % v/v). The supernatant from the centrifuged sample mixture was transferred in to a 15 mL of measuring cylinder. The residue was then re-suspended in 5 mL of MeOH:H_2_O (80:20, %v/v) and gently vortexed for an additional 30 min, followed by centrifugation. The supernatant was combined with the initial extract and the volume of the combined supernatant was adjusted to 10 mL with the extraction solvent. The methanolic extracts of *Awaze* paste was then tested for antioxidant activity using the 2,2-diphenyl-1-picrylhydracyl (DPPH) radical assay method as described [38,39]. For each concentration of methanol extract of *Awaze* paste (20, 40, 60, 80, 100 μL), 5 mL of a 0.1 m M methanol solution of DPPH radical was added to each formulation and concentration. The tubes containing *Awaze* paste sample solutions were allowed to stand at 27 °C for 20 min. The sample solution absorbance was recorded at 517 nm in a UV–Vis spectroscopy (Shimadzu UV–Vis Spectroscopy 1900). The IC50 value was determined as the amount of ethanolic extract necessary to decrease the initial DPPH radical concentration by 50 %. Antioxidant activity was determined according to the equation provided below (8):

(8) $$\text{Antioxidant}\;\text{activity}\;\left(\%\right)=\left(\frac{\text{Absorbance}\;\text{control}\;–\text{Absorbance}\;\text{sample}}{\text{Absorbance}\;\text{control}}\right)\;x\;100$$

#### 2.8.5 Iron (Fe) and zinc (Zn) content of *Awaze* paste

The mineral content (Fe and Zn) of the formulated *Awaze* paste and control sample were determined using AAS (Model: 240 FS, Agilent Series, America) following the method [32]. A 0.5 g *Awaze* paste sample was digested in a muffle furnace at 550 °C for 3 h until the residue ash turned white. The ashed *Awaze* paste sample was then digested using concentrated HCl. The digested *Awaze* paste sample was diluted to 100 mL for iron (Fe) and zinc (Zn) analysis. The Iron (Fe) and zinc (Zn) content of the formulated *Awaze* paste was obtained following the formula provided below (9):

(9) $$\text{Iron}\;\left(\text{Fe}\right)\;\text{and}\;\text{zinc}\;\left(\text{Zn}\right)\;\text{content}\;\left(\text{ppm}\right)=\frac{\left(\mathrm{conc}.\;\mathrm{AAS}-\mathrm{blank}\right)\times \mathrm{Volume}\;\mathrm{of}\;\mathrm{sample}\times \mathrm{dilution}\;\mathrm{factor}\;}{\mathrm{weight}\;\mathrm{of}\;\mathrm{sample}\;}$$

Where conc. AAS= Concentration reading of the sample from atomic absorption spectroscopy.

### 2.9 Rheological and Texture property of *Awaze* paste

#### 2.9.1 Viscosity

A 3 g of *Awaze* paste sample was dissolved in 5 mL of distilled water. The viscosity of *Awaze* paste solution was then determined using a Brookfield Viscometer (model Lv-3, Middleboro, MA 02346, USA) set at 60 rpm and a temperature of 25 °C [40].

#### 2.9.2 Texture of *Awaze* paste

The hardness of *Awaze* paste sample was measured using TPA (model: TA. XT. Plus, Stable Micro Systems, UK), following the method [41]. A P-2 cylindrical probe with a 2 mm diameter was used, and pre-test speed and test speed were set at 2 mm/s and 1 mm/s, respectively. Compression distance was set at 5 mm, with a load cell of 50 kg. Two compression were applied consecutively to the *Awaze* paste samples, and the hardness was measured.

### 2.10 Microbial profile

The total bacterial count, lactic acid bacteria count, yeast and mold count, and total coliform count were detected using the official method [32]. Samples of 25 g of *Awaze* paste were liquefied with 225 mL of 0.1 % peptone water. Serial dilution was performed by taking 1 mL from the diluted *Awaze* paste sample, and diluted samples (1 mL) were applied to the media surface using the pouring technique. Duplicate plates were prepared in all cases. The total bacterial count was estimated by applying PCA and incubating at 30 °C for 48 h. PDA was used to isolate yeast and mold, incubating at 25 °C for 48 h. LAB were estimated by using MRS agar and incubated at 30 °C for 48 h. Coliform were estimated by pouring VRBA and incubated at 35 °C for 24 h. Plates containing distinct colonies were selected and counted using a colony counter (Model: Scan 300, Inter Science). The microbial load was determined following the method [42]. The microbial load of *Awaze* paste was obtained according to the equation provided below (10):

(10) $$N=\frac{\sum n}{S\times d}$$

Where N = total count of bacteria, yeast and mold, lactic acid bacteria of *Awaze* sample, n = average number of bacteria, yeast and mold, lactic acid bacteria colony from different dilution, S = volume of sample for plating (mL), and d = dilution factor of *Awaze* paste sample.

### 2.11 Formula cost and yield percentage of the formulated *Awaze* paste

The cost and yield percentage of the formulated *Awaze* paste were determined using the formula [43]. The cost of formula and percentage of the formulated *Awaze* paste determined according to equation provided below (11 and 12):

(11) $$\text{Formula}\;\text{cost}=\frac{\text{Cost}\;\text{per}\;\text{kg}\;x\%\;\text{in}\;\text{formula}}{100}$$

(12) Yieldpercent=(EPQ/APQ)x100

Where EPQ = edible portion quantity and APQ = as purchased quantity.

### 2.12 Data analysis

Statistical analysis of the formulation and control sample was done using SPSS software (IBM, Chicago, IL, USA). For the formulation experiment Design Expert software version 13.0.1 was used. The individual effects of ingredients (spices) were illustrated using contour plots. ANOVA and the Tukey test were employed to determine the least significant difference among the formulations at (P < 0.05).

## 3 Result and discussion

### 3.1 Fitting of the model

Table 1 provides details on the mean porosity, particle size, pH, antioxidant activity, crude protein, zinc (Zn) content and iron (Fe) content of *Awaze* paste influenced by RP, GA, RO, and GI. Residual plots were created to assess the goodness of fit of the model. The best model exhibits an adequacy precision greater than 4, high predicted R-squared (R^2^), lack of fit (P > 0.05), low standard deviation, and low predicted sum of squares, which is considered desirable for the best fit to the model [44] as shown in Table 1. The linear model was identified as the best fit for zinc (Zn) content, while the quadratic model was identified as the best fit for antioxidant activity and particle size. The special cubic model was found to be the best fit for porosity, pH, crude protein content, and iron (Fe) content with R^**2**^ (0.93, 0.99, 0.97, and 0.99) respectively (Table 1). Therefore, the mixture model could be utilized to explore the design space.

**Table 1:** Effect of RP, GA, RO, and GI on the physicochemical quality of *Awaze* paste.

Response	model	R^2^	Lack of fit (p > 0.05)	Adequacy precision	F-value
Porosity	special cubic	0.93	0.62	3.21	1.18
Particle size	quadratic	0.87	0.06	7.92	4.05
pH	special cubic	0.99	0.1	22.4	56.39
Antioxidant activity	quadratic	0.72	0.34	4.76	1.5
Crude protein	special cubic	0.97	0.46	5.05	2.49
Iron (Fe)	special cubic	0.99	0.2	11.48	8.5
Zinc (Zn)	linear	0.47	0.06	5.39	3.29

Moreover, high F-values ranged from 1.18 to 56.39 and coefficients (R^**2**^) ranged from 0.47 to 0.99 ensuring a satisfactory fit of the proposed model to represent the actual relationship between the dependant and independent variables. The interaction between the amounts of RPxGI, ROxGI, and GAxGI in *Awaze* paste showed a significant negative effect only on particle size (F = 4.05, p = 0.02) as shown in Table 2. Therefore, this proposed mathematical model is valid and convenient for predicting the porosity, particle size, pH, antioxidant activity, crude protein, iron (Fe), and zinc (Zn) content of *Awaze* paste prepared under any combination of RP, GA, RO, and GI.

**Table 2:** Interaction effect of the independent variables (RP,GA,RO, and GI).

Response	Interaction (p value)									
	RP x GA	RPxRO	RPxGI	GAxRO	GAxGI	ROxGI	RPxGAxRO	RPxGAxGI	RPxROxGI	GAxROxGI
Porosity	0.51	0.8	0.47	0.34	0.52	0.83	0.56	0.26	0.6	0.4
Particle size	0.23	0.06	0.02	0.37	0.03	0.02	NIE	NIE	NIE	NIE
pH	0.6	0.11	0.08	0.21	0.12	0.52	0.24	0.05	0.06	0.34
Antioxidant activity	0.64	0.07	0.11	0.62	0.17	0.15	NIE	NIE	NIE	NIE
Crude protein	0.38	0.29	0.49	0.3	0.35	0.3	0.87	0.96	0.4	0.23
Iron (Fe)	0.28	0.27	0.24	0.09	0.39	0.24	0.18	0.10	0.23	0.12
Zinc (Zn)	NIE	NIE	NIE	NIE	NIE	NIE	NIE	NIE	NIE	NIE

NIE = no interaction effect, RP = red pepper, GA = garlic, RO = red onion, GI = ginger.

### 3.2 Effect of RP, GA, RO and GI on proximate composition of *Awaze* paste

The proximate composition of *Awaze* paste showed a significant difference (p < 0.05) between the formulations for moisture, crude protein, crude fat, crude fiber, total ash, total carbohydrate content, and energy value of the samples (Table 3). The moisture content of *Awaze* paste ranged from 4.66 % to 12.24 %. The highest moisture content (10.78 %) was obtained from F11 (74.39:13.33:5.0:7.28 RP, GA, RO, GI), while the lowest moisture content (4.6 %) was for the control sample. This difference may be due to the freshness of the prepared *Awaze* paste and the long storage of the control sample. The crude protein of the product ranged from 6.18 % to 16.22 %. The highest crude protein (16.22 %) was observed for F6 (76.39:10:8.6:5.01 RP, GA, RO, GI), while the lowest crude protein content, 6.18 %, was for the control sample.

**Table 3:** Proximate composition of formulated *Awaze* paste and control (household made *Awaze* paste).

Formula	Food composition of *Awaze* paste (%)						
	Moisture content	Crude protein	Crude fat	Total ash	Crude fiber	Total Carbohydrate	Energy (kcal.)
F1	9.15 ± 0.17 ^def^	15.31 ± 0.47 ^ab^	10.75 ± 0.21^b^	8.67 ± 0.22 ^de^	18.86 ± 0.52^h^	52.58 ± 0.78 ^cd^	307.06 ± 1.24^b^
F2	10.19 ± 0.14 ^bc^	11.69 ± 0.27 ^cd^	8.60 ± 0.28^d^	6.89 ± 0.03^h^	24.22 ± 0.33 ^ef^	50.10 ± 0.72^c^	277.81 ± 0.36 ^gh^
F3	8.28 ± 0.35 ^fg^	12.48 ± 0.68 ^bcd^	9.65 ± 0.35^c^	6.87 ± 0.04^h^	14.60 ± 0.54^i^	60.61 ± 0.59^a^	329.27 ± 0.83^a^
F4	10.72 ± 0.1^b^	16.13 ± 0.35^a^	12.70 ± 0.14^a^	8.25 ± 0.08 ^ef^	21.35 ± 0.21^g^	46.98 ± 0.54 ^fg^	302.22 ± 0.90 ^bc^
F5	12.24 ± 0.13^a^	11.55 ± 0.19 ^cd^	12.20 ± 0.00^a^	9.26 ± 0.06 ^bc^	18.68 ± 0.27^h^	47.63 ± 0.34 ^cde^	300.34 ± 1.36 ^bcd^
F6	7.75 ± 0.13 ^gh^	16.22 ± 0.52^a^	12.60 ± 0.28^a^	8.80 ± 0.04^d^	29.06 ± 0.48^a^	41.79 ± 0.93^h^	280.56 ± 1.17 ^fg^
F7	7.16 ± 0.01^h^	11.72 ± 0.23 ^cd^	12.40 ± 0.14^a^	7.79 ± 0.03^g^	27.50 ± 0.01 ^abc^	45.16 ± 0.16 ^ef^	292.24 ± 0.63 ^de^
F8	10.14 ± 0.06 ^bcd^	12.20 ± 0.37 ^cd^	8.40 ± 0.14^d^	7.90 ± 0.08 ^fg^	24.16 ± 0.46 ^ef^	49.42 ± 0.46 ^cd^	273.26 ± 0.57 ^ghi^
F9	8.10 ± 0.42 ^gh^	10.73 ± 0.18^d^	10.55 ± 0.35^b^	9.94 ± 0.06^a^	22.64 ± 0.47 ^fg^	48.78 ± 0.48^c^	290.07 ± 2.11 ^ef^
F10	9.49 ± 0.13 ^cde^	13.66 ± 0.8 ^abc^	8.55 ± 0.07^d^	7.56 ± 0.07^g^	27.55 ± 0.61 ^abc^	46.85 ± 0.48 ^ef^	264.35 ± 1.26^i^
F11	10.78 ± 0.32^b^	11.10 ± 1.95 ^cd^	12.15 ± 0.07^a^	8.84 ± 0.02 ^cd^	27.90 ± 0.83 ^ab^	40.34 ± 1.06^g^	270.73 ± 1.89 ^hi^
F12	9.30 ± 0.24 ^cde^	13.03 ± 0.96 ^bcd^	8.25 ± 0.07 ^de^	9.40 ± 0.03^b^	22.14 ± 0.23^g^	50.91 ± 0.06^c^	277.89 ± 0.39 ^gh^
F13	9.24 ± 0.04 ^cdef^	13.64 ± 0.54 ^abc^	7.50 ± 0.14^e^	6.32 ± 0.05^i^	19.39 ± 0.10^h^	57.56 ± 0.06^b^	297.75 ± 1.04 ^bcde^
F14	8.54 ± 0.47 ^efg^	13.66 ± 0.37 ^abc^	12.05 ± 0.35^a^	7.86 ± 0.04 ^fg^	25.12 ± 0.28 ^de^	46.44 ± 0.44 ^ef^	294.23 ± 1.94 ^cde^
F15	9.73 ± 0.38 ^cd^	12.86 ± 1.30 ^bcd^	9.05 ± 0.07 ^cd^	7.81 ± 0.02^g^	26.58 ± 0.49 ^bcd^	46.84 ± 0.01 ^def^	268.82 ± 0.69 ^hi^
Control	4.66 ± 0.16^i^	6.18 ± 0.20^e^	5.70 ± 0.14^f^	7.82 ± 0.308^g^	25.90 ± 0.14 ^cde^	50.28 ± 0.24^a^	277.16 ± 1.03 ^gh^

F = formulation, Result = mean standard deviation. Values with different lowercase letters in the same column are significantly different (P < 0.05).

Increasing the ratio of RP and GI increases the crude protein content of *Awaze* paste as shown in contour plot (Fig. 2e). The total ash content of the formulated *Awaze* paste and the control sample ranged from 6.32 % to 9.94 %. The crude fiber content of formulated paste sample ranged from 16.86 % to 29.06 %.The total carbohydrate content and the energy values of *Awaze* paste ranged from 40.34 % to 60.61 % and 264.35 kcal. to 329.27 kcal , respectively. The observed differences between the formulations and the control sample for crude fat, crude protein, crude fiber, total ash, total carbohydrate content, and energy value are probably due to the difference in the storage time, raw materials, and preparation method.

A previous study on Korean red pepper paste (*gochujang)* reported crude fat, crude protein, total ash, total carbohydrate, and energy content ranging from 0.3 % to 3.1 %, 3.4 %–9.3 %, 5.2 %–21.1 %, and 129 kcal. to 247.97 kcal, respectively [45], which was in agreement with the current result for total ash content. However, there were differences in crude fat, crude protein, total ash, total carbohydrate, and energy content, which may be attributed to differences in raw materials and processing methods. The proximate compositions (except moisture and total carbohydrate) of this study were higher than the findings reported in a study on garlic and ginger-enhanced ogi paste [46], The reported values for crude protein, crude fat, crude fiber, total ash, moisture, and total carbohydrate were in the range of 9.83 %–10.92 %, 2.34 %–3.06 %, 1.47 %–2.42 %, 0.28 %–1.44 %, 16.32 %–25.67 %, and 59.24 %–66.95 %, respectively. A similar study on onion paste reported comparable results to the current study [47]**.**

### 3.3 Effect of RP, GA, RO, and GI on the techno-functional properties of composite powder

The techno-functional properties of the composite powder formulations are presented in Table 4. The water absorption capacity of the formulations ranged from 1.59 % to 2.36 %. Previous studies have reported the water absorption capacity of RP (1.86 %) and GA (2.51 %) [6]. Formulations F2 (67.28:19.37:8.28:5.07 RP, GA, RO, GI) and F4 (68.84:13.99:12.16:5.01 RP, GA, RO, GI) had the highest water absorption capacity, possibly due to the higher proportion of GA and RO in the mixture, while formulation F12 (63.27:24.68:7.03:5.02 RP, GA, RO, GI) had the lowest water absorption capacity (1.59 %). Bulk density, tapped density, porosity, and flowability are important parameters for saving packaging material, transportation, and processing of raw materials during manufacturing [48]. The bulk density ranged from 351.9 kg/m^3^ to 409.4 kg/m^3^, and tapped density ranged from 436 kg/m^3^ to 547 kg/m^3^ in the formulations. The highest bulk density (409.4 kg/m^3^) was obtained for F10 (65.27:10:17.7:7.03 RP, GA, RO, GI). The highest tapped density (547 kg/m^3^) was found for F6 (76.39:10:8.6:5.01 RP, GA, RO, GI). These may be due to the highest proportion of GA and RO in the formulation.

**Table 4:** Techno-functional property of the formulated composite powder and total soluble solid of *Awaze* paste from RP, GA, RO and GI.

Formula	Functional property of composite powders and TSS of *Awaze* paste						
	Water absorption capacity (%)	Bulk density (kg/m^3^)	Tapped Density (kg/m^3^)	Porosity (%)	Hausenr ratio (HR)	Carr's index (CI)	Total soluble solid (^o^Brix)
F1	1.69 ± 0.08 ^bc^	375.9 ± 0.00^b^	466 ± 0.5 ^cd^	19.4 ± 0.88 ^ab^	1.2 ± 0.01 ^ab^	19.4 ± 0.88 ^ab^	7.2 ± 0.14^b^
F2	2.36 ± 0.20^a^	401 ± 0.00^a^	470 ± 1.03 ^bcd^	14.7 ± 1.89 ^ab^	1.2 ± 0.03 ^ab^	14.7 ± 1.89 ^ab^	4.05 ± 0.07^f^
F3	2.20 ± 0.07 ^ab^	409.4 ± 1.18^a^	470 ± 1.03 ^bcd^	12.9 ± 0.59 ^ab^	1.2 ± 0.01^b^	12.9 ± 0.59 ^ab^	3.7 ± 0.14^f^
F4	2.36 ± 0.20^a^	358.1 ± 0.60^b^	436 ± 0.90^d^	17.9 ± 0.3 ^ab^	1.2 ± 0.01 ^ab^	17.9 ± 0.3 ^ab^	6.35 ± 0.07 ^cd^
F5	1.93 ± 0.11 ^abc^	355.9 ± 0.30^b^	470 ± 1.03 ^bcd^	24.3 ± 1.04^a^	1.3 ± 0.01 ^ab^	24.3 ± 1.04^a^	6.7 ± 0.14 ^bc^
F6	1.70 ± 0.07 ^bc^	375.9 ± 0.00^b^	547 ± 0.00^a^	31.3 ± 0.00^a^	1.5 ± 0.00^a^	31.3 ± 0.00^a^	3.85 ± 0.21^f^
F7	1.74 ± 0.02 ^bc^	360.3 ± 0.91^b^	474 ± 1.58 ^bcd^	24 ± 0. 61^a^	1.3 ± 0.01 ^ab^	24 ± 0. 61^a^	6.3 ± 0.14 ^cd^
F8	1.73 ± 0.02 ^bc^	351.9 ± 0.87^b^	501 ± 0.00 ^bc^	29.8 ± 1.74^a^	1.4 ± 0.04 ^ab^	29.8 ± 1.74^a^	8.35 ± 0.07^a^
F9	1.76 ± 0.08 ^bc^	358.1 ± 0.60^b^	514 ± 1.87 ^ab^	30.3 ± 3.7^a^	1.4 ± 0.08 ^ab^	30.3 ± 3.7^a^	5.2 ± 0.14^e^
F10	2.05 ± 0.15 ^abc^	409.4 ± 1.18^a^	470 ± 1.03 ^bcd^	12.9 ± 4.44 ^ab^	1.2 ± 0.06^b^	12.9 ± 4.44 ^ab^	6.75 ± 0.07 ^bc^
F11	2.03 ± 0.06 ^abc^	401 ± 0.00^a^	514 ± 1.87 ^ab^	22 ± 2. 83^a^	1.3 ± 0.05 ^ab^	22 ± 2. 83^a^	3.5 ± 0.14 ^fg^
F12	1.59 ± 0.09^c^	358.1 ± 0.60^b^	510 ± 1.22 ^abc^	29.8 ± 0.5^a^	1.4 ± 0.01 ^ab^	29.8 ± 0.5^a^	6.5 ± 0.14^c^
F13	1.97 ± 0.37 ^abc^	353.8 ± 0.00^b^	466 ± 0.51 ^cd^	24.1 ± 0.83^a^	1.3 ± 0.01 ^ab^	24.1 ± 0.83^a^	3.1 ± 0.14^g^
F14	1.63 ± 0.18 ^bc^	355.9 ± 0.30^b^	470 ± 1.03 ^bcd^	24.3 ± 1.04^a^	1.3 ± 0.01 ^ab^	24.3 ± 1.04^a^	4.85 ± 0.21^e^
F15	2.13 ± 0.04 ^abc^	401 ± 0.00^a^	470 ± 1.03^bcd^	14.7 ± 1.89 ^ab^	1.2 ± 0.03 ^ab^	14.7 ± 1.89 ^ab^	4.8 ± 0.14^e^
Control	NI	NI	NI	NI	NI	NI	5.9 ± 0.14^d^

F = formulation, NI = not important, Result = mean standard deviation. Values with different lowercase letters in the same column are significantly different (P < 0.05).

The porosity of the formulations ranged from 12.9 % to 31.3 %. The lowest porosity was obtained for F3 (70.13:10:9.87:10 RP, GA, RO, GI), with the lowest porosity value being 12.9 %. The formulation F6 (76.39:10:8.6:5.01 RP, GA, RO, GI) showed a porosity of 31.3 %, which was the highest porosity among the formulations; this could be due to the larger particle size of RP. The results of the current study were similar to the reported result [49]. As the proportion of GA and GI increases, the porosity was significantly increased (p < 0.05) (Fig. 2a). As the mixing ratio of RP and RO increases, the porosity significantly decreased. Therefore, we suggest that a significant difference in porosity is due to the difference in the proportion of ingredients (RP, GA, RO and, GI) in the formulation.

Flowability is expressed as the free flow of powders during food processing, handling, and transportation. Flowability of powders can be expressed as Hausen ratio (HR) and Carr's index (CI) [50]. Powders having (HR < 1.34) and (CI < 25 %) can be considered as better in flowability. In the current study, F6 (76.39:10:8.6:5.01 RP, GA, RO, GI) had poor flow-ability with an HR value of 1.5 and CI value of 31.3 as shown in Table 3. Powder flowability is directly related to the particle size uniformity of powders [51]. Powder flowability is an important property in commercial applications such as storage, transportation, manufacturing, and processing of food products [52]. Similar results were studied for red pepper paste that was made only from red pepper powder, with values of HR (1.48) and CI (32 %) [53].

### 3.4 Effect of RP, GA, RO and GI on particle size and surface area of the composite powder

The particle size of the formulated composite powder of *Awaze* paste was reported as D_10_, D_50_, and D_90_ percentiles (Table 5). The particle size of the formulated composite powder of *Awaze* paste showed a wide range, with D_10_ ranged from 13.25 μm to 25.05 μm, D_50_ from 91.5 μm to 172 μm, and D_90_ from 373.5 μm to 558.5 μm. Formulation F2 (67.28:19.37:8.28:5.07 RP, GA, RO, GI), had a D_50_ value of 172 μm and a D_90_ value of 558 μm, showing a higher particle size compared to *Awaze* paste with lower GA and RP proportions. This difference may be attributed to the larger particle size of GA and RP powders. Significant difference at (P < 0.05) were observed for specific surface area and volume-weighted mean diameter among the formulation, with specific surface areas ranging from 58.45 m^2^/g to 111.8 m^2^/g.

**Table 5:** Particle size of the composite powder (D_10, 50, 90_), and pH, and titratable acidity of *Awaze* paste from RP, GA, RO and GI.

Particle size, surface area, pH and Titratable							
Formula	D_10_(μm)	D_50_(μm)	D_90_(μm)	Specific surface area (m^2^/g)	Volume weighted mean diameter (D 43) (μm)	pH	Titratable acidity (%)
F1	19.70 ± 0.28^b^	141.00 ± 1.41^c^	472.00 ± 4.14 ^cd^	90.10 ± 3.58^e^	168.00 ± 1.41 ^fg^	5.65 ± 0.07 ^ab^	0.19 ± 0.01^a^
F2	16.65 ± 0.21 ^de^	172.00 ± 5.66^a^	558.50 ± 2.02^a^	92.33 ± 0.14 ^de^	230.50 ± 3.19^a^	5.55 ± 0.07 ^abc^	0.23 ± 0.05^d^
F3	17.50 ± 0.5 ^cd^	120.00 ± 4.24 ^ef^	388.50 ± 3.19^h^	89.52 ± 0.32^e^	174.50 ± 2.36 ^ef^	5.75 ± 0.07^a^	0.17 ± 0.04^f^
F4	16.50 ± 0.57 ^de^	121.50 ± 4.95 ^de^	449.00 ± 0.00 ^def^	103.65 ± 0.21 ^bc^	189.00 ± 2.83 ^cde^	5.75 ± 0.07^a^	0.2 ± 0.04^e^
F5	18.25 ± 0.35^c^	160.00 ± 1.4^b^	505.50 ± 0.71^b^	88.12 ± 0.95^e^	218.50 ± 0.71 ^ab^	5.75 ± 0.07^a^	0.23 ± 0.04^d^
F6	17.70 ± 0.14 ^cd^	137.00 ± 1.4^c^	490.00 ± 4.24 ^bc^	93.78 ± 3.05 ^de^	203.50 ± 2.12 ^bc^	5.2 ± 0.00 ^de^	0.26 ± 0.05^c^
F7	15.25 ± 0.07 ^efg^	121.50 ± 4.95 ^cd^	505.50 ± 3.54^b^	100.60 ± 0.28 ^bc^	210.00 ± 0.00^b^	5.25 ± 0.07 ^cde^	0.30 ± 0.04 ^ab^
F8	15.65 ± 0.35 ^ef^	91.55 ± 0.49^g^	377.00 ± 2.83^h^	105.85 ± 0.64 ^ab^	154.00 ± 2.66^g^	5.5 ± 0.14 ^abcd^	0.27 ± 0.05^c^
F9	16.40 ± 0.57 ^de^	117.50 ± 3.54 ^ef^	425.00 ± 5.66 ^fg^	98.19 ± 1.47 ^cd^	175.00 ± 2.66 ^ef^	5.45 ± 0.07 ^abcde^	0.19 ± 0.02^e^
F10	14.75 ± 0.21 ^fgh^	129.50 ± 2.12^c^	447.50 ± 2.12 ^def^	105.70 ± 1.70^b^	187.50 ± 0.71 ^de^	5.55 ± 0.07 ^abc^	0.31 ± 0.06^b^
F11	25.05 ± 0. 64^a^	111.50 ± 0.71 ^ef^	373.50 ± 4.95^h^	105.90 ± 1.41 ^ab^	108.50 ± 0.71^h^	5.35 ± 0.07 ^bcde^	0.19 ± 0.02^e^
F12	14.75 ± 0.07 ^fgh^	136.50 ± 2.12^c^	452.50 ± 0.71 ^de^	58.45 ± 1.36^f^	193.50 ± 0.71 ^cd^	5.55 ± 0.07 ^abc^	0.22 ± 0.04^d^
F13	14.65 ± 0.21 ^fgh^	120.50 ± 0.71 ^de^	427.50 ± 2.12 ^fg^	103.55 ± 0.35 ^bc^	181.00 ± 1.41 ^def^	5.55 ± 0.07 ^abc^	0.19 ± 0.0 e
F14	13.65 ± 0.07^h^	108.00 ± 1.41^f^	421.00 ± 2.83^g^	104.90 ± 0.99^b^	170.50 ± 0.71^f^	5.7 ± 0.14^a^	0.19 ± 0.03^e^
F15	13.85 ± 0.07 ^gh^	120.50 ± 0.71 ^de^	433.00 ± 5.66^efg^	111.80 ± 0.85^a^	169.00 ± 4.24 ^fg^	5.35 ± 0.07 ^bcde^	0.32 ± 0.04^e^
Control	NI	NI	NI	NI	NI	5.15 ± 0.05^e^	0.16 ± 0.03^f^

F = formulation, NI = not important, Result = mean ± standard deviation. Values with different lowercase letters in the same column are significantly different (P < 0.05).

The highest volume-weighted mean diameter (230.5 μm) was observed for F2 (67.28:19.37:8.28:5.07 RP, GA, RO, GI), while the lowest value (108.5 μm) was measured for F11. This difference indicated that F11 (74.39:13.33:5:7.28 RP,GA,RO,GI), had the finest powder among all the formulations. Increasing the proportion of RP and GI led to an increase in the particle size of the composite powder, while an increase in the proportion of GA and RO decreased the particle size (D10) of the composite powder as shown in contour plot in (Fig. 2b).

### 3.5 Effect of RP, GA, RO and GI on total soluble solid, pH and titrable acidity of *Awaze* paste

Formulations significantly affected the total soluble solid (TSS) of *Awaze* paste at (p < 0.05). F8 (60:15:20:5.0 RP, GA, RO, GI) had the highest TSS value. This may be due to the highest mixing ratio of RO in the formulation. Optimal pH is one of the pre-requisites for the quality of foods and affects the occurrence of several biochemical activities [45]. The formulations exhibited pH ranged from 5.15 to 5.75. The lowest pH value was found in the control sample (5.15), while the highest pH value (5.75) were obtained for F3 (70.13:10:9.87:10 RP, GA, RO, GI), F4 (68.84:13.99:12.16:5.01 RP, GA, RO, GI), and F5 (60:30:5:5 RP, GA, RO, GI). However, the pH value of the formulations and control sample were statistically significantly different (Table 5). Foods with a pH value greater than 4.6 are categorized as low acidic foods, and with a pH below 4.6 are acidic foods [54]. All formulations had a pH value lower than 5.2. This may be due to the non-fermented (fresh) nature of the formulation.

The low pH value of the control sample (5.15) that was household-made may be due to the fermentation of the product as a result of long storage time. A previous study report showed a pH range of 4.59–4.79 for red pepper paste [55]. The present study observed similar pH to the report of [56], that was conducted on several laboratory-made red pepper paste. The pH content of *Awaze* paste was increased with an increase in the proportion of RP and RO as shown (Fig. 2c).

Titratable acidity (TA) indicates the extent of fermentation and acidity in *Awaze* paste. The titratable acidity of formulations ranged from 0.15 % to 0.32 %. F15 (60:14.81:15.2:9.99 RP, GA, RO, GI) had the highest titratable acidity of 0.32 %. TA increased with more GA and less RP. A study on red pepper and tomato pastes reported titratable acidity ranging from 1.12 % to 2.59 % [57], which is lower than the current results.

### 3.6 Effect of RP, GA, RO and GI on antioxidant activity of *Awaze* paste

The antioxidant activity of the formulated *Awaze* paste towards DPPH radical was measured at different concentrations (Table 6). There was a significant difference (p < 0.05) between the formulations in antioxidant activity. The antioxidant activity ranged from 11.16 % to 62.5 % for formulations at different concentrations (20, 40, 60, 80, 100 μL), and the antioxidant activity increased with an increase in concentration from 20 to 100 μL (Table 6). The highest percentage of inhibition, 54.13 %, was found for F1 (60: 20.2:12.01:7.77 RP,GA, RO,GI), while the lowest percentage of inhibition, 34.24 %, was obtained for F4 (68.84:13.99:12.16:5.01 RP, GA, RO, GI) at a low concentration (20 μL).These findings showed that the formulation affects the antioxidant activity of *Awaze* paste.

**Table 6:** Effect of concentration (20, 40, 60, 80, 100 μL) on antioxidant activity of *Awaze* paste and control sample.

Formula	Antioxidant activity				
	Concentration				
	20 (μL)	40 (μL)	60 (μL)	80 (μL)	100 (μL)
F1	54.14 ± 2.14 ^ab^	56.49 ± 0.21 ^ab^	54.45 ± 1.48 ^cdef^	57.55 ± 1.33 ^cd^	63.32 ± 0.22 ^ab^
F2	42.44 ± 1.09 ^de^	47.00 ± 0.09 ^de^	56.15 ± 0.56 ^cdef^	59.22 ± 1.0 ^bc^	62.79 ± 0.43 ^ab^
F3	48.48 ± 1.91 ^bcd^	44.83 ±1.06 ^ef^	55.81 ± 0.24 ^def^	47.26 ± 0.49^i^	58.24 ± 1.14 ^cd^
F4	34.24 ± 1.06^f^	56.15 ± 1.34 ^ab^	58.69 ± 0.2 ^bc^	48.02 ± 0.04 ^hi^	59.72 ± 1.63 ^bc^
F5	55.88 ± 0.64^a^	41.57 ± 1.55^g^	56.57 ± 0.53 ^bcdef^	58.88 ± 0.7 ^bc^	63.93 ± 1.07^a^
F6	48.4 ± 0.37 ^bcd^	55.05 ± 0.21 ^bc^	45.75 ± 0.27^h^	53 ± 0.11^e^	62.6 ± 1.44 ^ab^
F7	44.99 ± 0.8 ^cde^	49.62 ± 0.38^d^	54.71 ± 0.05^f^	61.35 ± 0.21 ^ab^	64.42 ± 0.37^a^
F8	48.1 ± 2.36 ^bcd^	56.9 ± 1.95 ^ab^	55.5 ± 0.21 ^ef^	59.68 ± 0.00 ^bc^	53.76 ± 1.82^e^
F9	51.1 ± 1.82 ^abc^	59.15 ± 0.21^a^	58.47 ± 0.89 ^bcd^	59.41 ± 0.05 ^bc^	58.61 ± 0.1 ^cd^
F10	48.52 ± 0.96 ^bcd^	55.08 ± 0.37 ^bc^	56.3 ± 0.16 ^cdef^	61.2 ± 0.96 ^ab^	58.88 ± 1.84 ^cd^
F11	33.94 ± 1.4^f^	56.19 ± 0.2 ^ab^	57.85 ± 0.11 ^bcde^	62.52 ± 0.16^a^	61.92 ± 1.13 ^abc^
F12	31.51 ± 0.86^f^	54.59 ± 0.22 ^bc^	61.96 ± 0.32^a^	55.73 ± 0.22^d^	59.79 ± 0.26 ^bc^
F13	44.65 ± 0.18 ^cde^	55.83 ± 0.06^b^	52.2 ± 0.05^g^	57.74 ± 0.91 ^cd^	58.69 ± 0.96 ^cd^
F14	48.02 ± 1.09 ^bcd^	42.97 ± 0.23 ^fg^	58.28 ± 0.1 ^bcde^	50.3 ± 0.26 ^gh^	58.73 ± 1.88 ^cd^
F15	40.54 ± 1.29^e^	42.86 ± 1.31 ^fg^	51.67 ± 0.48^g^	52.12 ± 0.49 ^fg^	58.16 ± 1.07 ^cd^
Control	42.25 ± 0.58 ^de^	52.62 ± 1.07^c^	59.22 ± 0.16^b^	46.77 ± 0.07^i^	55.35 ± 2.2 ^de^

The highest percentage of inhibition in F1(60: 20.2:12.01:7.77 RP,GA, RO,GI) was because of the high proportion of RP and GA. This finding was in agreement with a study [58] that reported percentages of inhibition for Brazilian spices ranging from 30 to 90 %. Another study reported that the percentages of inhibition of hot red pepper paste ranged from 44 % to 90 % [8], which was higher than the current result. Increasing the proportion of RP, GA, and GI in the mixture enhances the percentages of inhibition of the formulated *Awaze* paste, as shown in the contour plot (Fig. 2d).

**Fig. 1:**
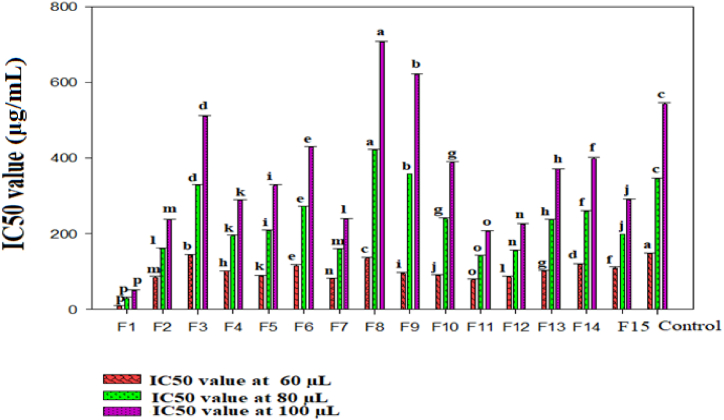
The IC50 value of the formulations at different concentrations (60 μL, 80 μL, and 100 μL).

The IC50 value in each formulation gave information regarding the quality and reactivity, indicating the amount of free radical scavenger compounds present in each formulations. The lower the IC50 value, the more potent the substance is at scavenging DPPH radical, indicating a higher antioxidant activity. The IC50 value was ranged from 8.5 μg/mL to 706.78 μg/mL at different concentrations as shown in (Fig. 1). Considering the parameters IC50 value obtained by the DPPH radical assay, F8 (60:15:20:5 RP, GA, RO, GI) had the highest IC50 value 421.26 μg/mL at 80 μL and 706.78 μg/mL at 100 μL respectively; it may be because of the higher mixing ratio of RO in the formulation. On the other hand, F1 (60: 20.2:12.01:7.77 RP,GA, RO,GI) had the lowest IC50 value 8.5 μg/mL, 29.88 μg/mL, and 51.11 μg/mL at 60 μL, 80 μL, and 100 μL respectively, meaning that compared to other formulation F1 extract can be considered a fast acting free radical scavenger. Previous study on onion, garlic and ginger reported IC50 value 2224 μg/mL, 41 μg/mL, and 19.1 μg/mL respectively [59]. In the other study, the IC50 values were found to range from 694.8 μg/mL to 1153.63 μg/mL for colored bell peppers [60]. Notably, both reports indicated higher IC50 values compared to the current study.

**Fig. 2:**
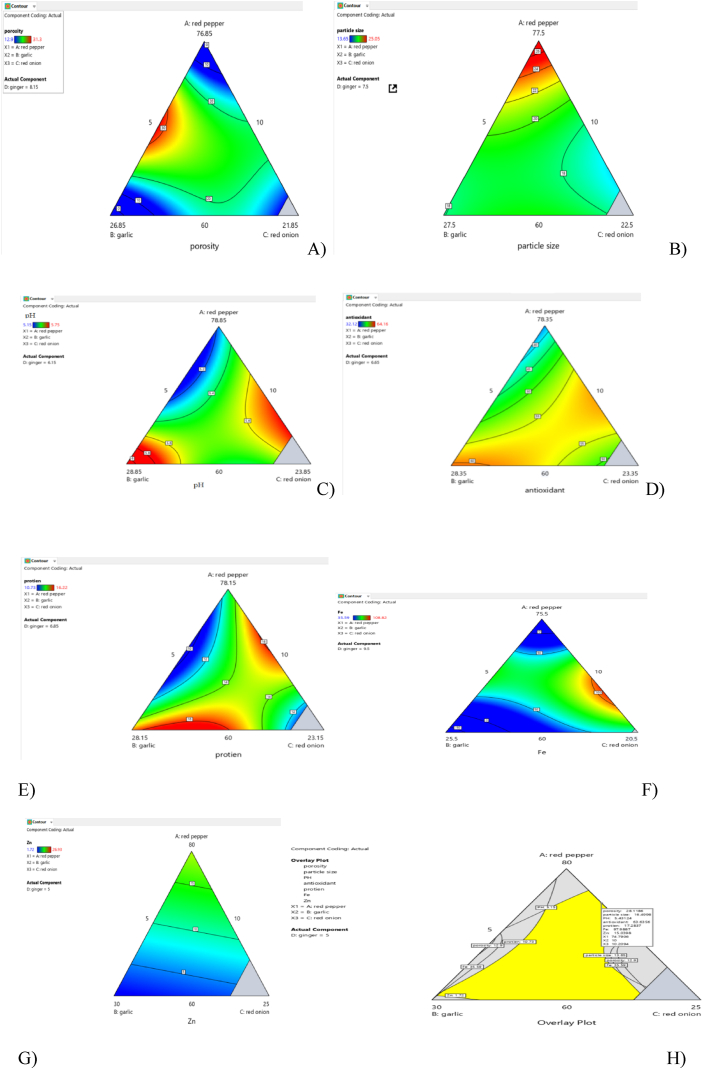
Effect of RP, GA, RO, GI on A) porosity, B) particle size (D_10_), C) pH, D) antioxidant activity, E) protein content, F) iron (Fe) content G) zinc (Zn) content H) Overlaid contour plot of formulated *Awaze* paste.

### 3.7 Effect of RP, GA, RO and GI proportion on iron (Fe) and zinc (Zn) content, rheological and textural property of *Awaze* paste

The iron (Fe) content, zinc (Zn) content, viscosity, and hardness of the formulated *Awaze* paste were significantly different (p < 0.05) (Table 7). The iron (Fe) and zinc (Zn) content of *Awaze* paste ranged from 36.54 mg/100g to 108.82 mg/100g and 1.72 mg/100g to 26.93 mg/100g, respectively. F6 (76.39:10: 8.6:5.01 RP, GA, RO, GI) had the highest Fe content (108.82 mg/100 g) and Zn content (26.93 mg/100 g). This may be attributed to the higher iron (Fe) and zinc (Zn) content of RP and RO [6]. The iron (Fe) and zinc (Zn) content of *Awaze* paste increased significantly, as the proportion of RP and GI increased in the mixture, as shown in the contour plot (2f and 2g).

**Table 7:** Mineral content, rheological and textural property of *Awaze* paste and Household made sample.

Formula	Mineral content		Rheological property	Textural property
	Fe (mg/100g)	Zn (mg/100g)	Viscosity (cps)	Hardness (g)
F1	65.08 ± 0.14^d^	3.26 ± 0.32^g^	102 ± 1.4 ^cd^	8.88 ± 0.19 ^fg^
F2	47.62 ± 0.59^h^	12.25 ± 0.12^b^	110 ± 2.83 ^bc^	8.48 ± 0.37^g^
F3	61.43 ± 0.51^f^	11.42 ± 0.08^c^	121 ± 2.83^a^	13.36 ± 0.77 ^def^
F4	49.22 ± 0.11^h^	4.52 ± 0.03^f^	76.5 ± 2.12 ^ghi^	11.35 ± 0.64 ^efg^
F5	35.59 ± 0.12^k^	1.86 ± 0.09 ^jk^	111.5 ± 0.7^b^	13.62 ± 1.22 ^de^
F6	108.82 ± 0.82^a^	26.93 ± 0.16^a^	89.5 ± 2.12 ^ef^	14.43 ± 0.69 ^de^
F7	62.82 ± 0.02 ^ef^	8.57 ± 0.27^d^	125.5 ± 2.12^a^	10.11 ± 0.55 ^efg^
F8	73.83 ± 0.01^b^	3.11 ± 0.08 ^gh^	93.5 ± 0.71^e^	16.33 ± 0.04^d^
F9	63.36 ± 0.64^e^	5.6 ± 0.12^e^	91.5 ± 2.12 ^ef^	12.57 ± 0.13 ^defg^
F10	45.37 ± 0.74^i^	1.72 ± 0.09^k^	104.5 ± 2.1 ^bc^	23.59 ± 1.29^c^
F11	59.61 ± 0.07^g^	3.59 ± 0.12^g^	78.5 ± 2.12^i^	28.68 ± 2.08^b^
F12	71.91 ± 0.08^c^	2.5 ± 0.01 ^hi^	71 ± 1.41 ^gh^	25.41 ± 1.73 ^bc^
F13	72.34 ± 0.11 ^bc^	3.58 ± 0.05^g^	68.5 ± 0.71 ^hi^	21.71 ± 0.24^c^
F14	36.54 ± 0.47^k^	3.51 ± 0.06^g^	76.5 ± 2.12 ^ghi^	15.96 ± 0.90^d^
F15	41.51 ± 0.29^j^	2.43 ± 0.08 ^ij^	95.5 ± 2.12 ^de^	55.09 ± 2.78^a^
Control	58.68 ± 0.48^g^	4.63 ± 0.13^f^	84.5 ± 3.50 ^fg^	14.12 ± 0.06 ^de^

The viscosity of the formulations and control sample ranged from 71 cps to 125.5 cps. The highest viscosity was found for F7 (64.55:15.46:10.17:9.82 RP, GA, RO, GI). The lowest viscosity values were found for formulation F12 (63.27: 24.68: 7.03: 5.02 RP, GA, RO, GI). This could be due to the higher mixing ratio of GI and RO, as GI and RO have higher viscosity compared to RP and GA as reported by Ref. [6]. The viscosity in the current results is higher than the reported viscosity values (13.24 cps–85.84 cps) of hot pepper-soya bean paste [61]. F15 (60:14.81: 15.2: 9.9 RP, GA, RO, GI) had the highest hardness value (55.09 g), which was due to the highest proportion of GA and GI.

### 3.8 Microbiological quality of formulated *Awaze* paste

**Fig. 3:**
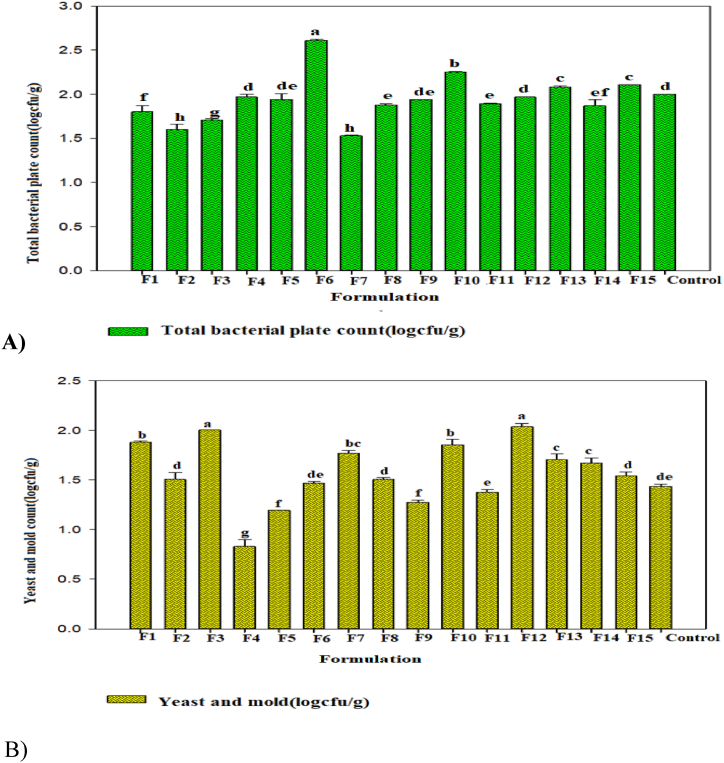
A) Total bacterial count and B) yeast and mold count of the formulated *Awaze* paste.

Microbiological analysis at zero time (without storage period) for the formulations showed that lactic acid bacteria and coliform were not detected in the fresh *Awaze* paste sample. The tested *Awaze* paste product, total bacterial plate count was ranged from 1.53 log cfu/g to 2.61 log cfu/g (Fig. 3a), and yeast and mold count in all formulations ranged from 0.83 log cfu/g to 2.04 log cfu/g (Fig. 3b). Considering 6 log cfu/g as the maximum limit of total bacterial count and 4 log cfu/g for yeast and mold, it resulted that the formulated *Awaze* paste is safe and acceptable according to international standards for food safety [42].

The microbial population in food samples is highly dependent on physicochemical, microbial profile, and the external environment of the raw material. The microbial population in the samples (*Awaze* paste), especially total bacteria count (<6 log cfu/g) and yeast and mold (<4 log cfu/g), indicated that the raw materials used for formulations were handled and dried in hygienic conditions. The findings from the present study for bacteria count, yeast, and mold were in agreement with the reported results [57,62]. Compared to the findings of [45] conducted on fermented red pepper paste (*gochujang*), reported total bacteria plate count, yeast, and mold were ranged from 2.79 log cfu/g to 8.73 log cfu/g and 1.56 log cfu/g to 7.15 log cfu/g, respectively, which is not in agreement to the current results.

### 3.9 Optimization

The optimum formula was determined by numerical and graphical optimization in *Awaze* paste samples prepared within the range comprising RP (60–90 %) , GA (10–30 %), RO (5–20 %), and GI (5–10 %). The optimum formula includes all the selected parameters at optimum values (porosity, particle size, pH, antioxidant activity, crude protein, iron (Fe) content, and zinc (Zn) content) as indicated in (Fig. 2h). The proportion of RP, GA, and GI were set to the range, while RO was set to the maximum. The antioxidant activity, crude protein, iron (Fe) content, and zinc (Zn) content were set to maximum, while the other parameters were set within the range. A high relative importance of “5” was given to the antioxidant activity, protein, iron (Fe) content, and zinc (Zn) content of formulations.

Based on numerical and graphical optimization, *Awaze* paste made from 74.79:10.0:10.20:5 RP, GA, RO, and GI, respectively, with high desirability (0.73) is the optimum formula for *Awaze* paste (Fig. 2h). The indicated region in Fig. 2h suggested that any point within this region represents an accepted formula in terms of the quality of *Awaze* paste. The optimum formula had antioxidant activity (63.6 %), crude protein content (17.28), iron (Fe) (98.06 mg/100 g), and zinc (Zn) content (15.04 mg/100 g), while the control sample had antioxidant activity (55.35 %), crude protein content (6.18), iron (Fe) (58.68 mg/100 g), and zinc (Zn) content (4.68 mg/100 g). The results indicated that the optimum formula is better in nutritional value compared to the control sample.

### 3.10 Formula cost and yield percentage of *Awaze* paste

The formula cost of *Awaze* paste is shown in Table 8. Accordingly, the cost of the optimized *Awaze* paste is 301.95 birr per kg (5.25 USD/kg), which is affordable to consumers. Because the cost is low compared to other formulated and manufactured food products available in supermarkets in Ethiopia. The yield percentage for the formulation was also found to be 84.61 % by considering GA, RO, and GI as wet spice (washed and peeled spice) from all the spices used to prepare the optimized *Awaze* paste. During formulation, the wet spices, GA, RO, and GI were washed and peeled, resulting in trim (loss) due to washing and peeling of the cover skin to be edible. Generally, the formula cost and yield percentage indicate a profitable product formulation, and as a result, any entrepreneur can start to manufacture the product using the optimum formula for *Awaze* paste.

**Table 8:** Formula cost of the formulated *Awaze* paste from the optimum formula.

Ingredient	Cost(Birr) per kg	Cost(Dollar) per kg	% in formulation	Cost (Birr)	Cost(Dollar)
Red pepper	300	5.22	74	222	3.8662487
Garlic	150	2.61	10	15	0.261233
Red onion	50	0.87	10.2	5.1	0.0888192
Ginger	120	2.09	5	6	0.1044932
Cardamom	200	3.48	5	10	0.1741553
Fenugreek	300	5.22	5	15	0.261233
White cumin	300	5.22	2.5	7.5	0.1306165
Basil	150	2.61	2.5	3.75	0.0653083
Black cumin	300	5.22	1.25	3.75	0.0653083
*Mekelesha*	200	3.48	1.25	2.5	0.0435388
Rue	200	3.48	0.1	0.2	0.0034831
Coriander	150	2.61	0.5	0.75	0.0130617
Rosemary	200	3.48	0.4	0.8	0.0139324
Thyme	400	6.97	0.4	1.6	0.0278649
Salt	40	0.70	20	8	0.139
Water	0.00	0.00	150	0.00	0.00
Total				301.95 Birr	5.25 Dollar

1USD = 57.42 Birr (current Dollar conversion to Birr in Ethiopia).

## 4 Conclusion

Red pepper significantly increases the total mineral content, iron (Fe) content, and zinc (Zn) content of *Awaze* paste. Garlic significantly improves the flowability, particle size, and antioxidant activity of *Awaze* paste. An acceptable *Awaze* paste was formulated with 74.79 % RP, 10 % GA, 10.2 % RO, and 5.0 % GI. The formulated *Awaze* paste is rich in antioxidant activity, crude protein, total ash content, iron (Fe) content, and zinc (Zn) content. The paste produced has a low microbial load, indicating that the product is shelf-stable. This study shows the potential of utilizing these locally produced spice raw materials and is important for new product development for household producers, cottage industry, and the spice industry. In the future, a morphological study and the influence of storage materials and methods on the physicochemical and microbiological quality of the optimized *Awaze* paste are highly recommended.

## Ethics declaration

Review or approval by an ethics committee was not needed for this study because the experiment is not dependent on human or animal subjects.

## Funding statement

This research received a grant from the 10.13039/501100004535Ethiopian Institute of Agricultural Research (10.13039/501100004535EIAR) for the PhD project work.

## Data availability statement

Data will be made available on request.

## CRediT authorship contribution statement

**Biadge Kefale:** Writing – review & editing, Writing – original draft, Visualization, Validation, Software, Resources, Methodology, Investigation, Funding acquisition, Formal analysis, Data curation, Conceptualization. **Mulugeta Admasu Delele:** Writing – review & editing, Writing – original draft, Visualization, Validation, Supervision, Software, Resources, Methodology, Investigation, Formal analysis, Data curation, Conceptualization. **Solomon Workneh Fanta:** Writing – review & editing, Writing – original draft, Visualization, Validation, Supervision, Software, Resources, Methodology, Investigation, Formal analysis, Data curation, Conceptualization. **Solomon Abate:** Writing – review & editing, Writing – original draft, Visualization, Validation, Supervision, Software, Resources, Methodology, Investigation, Formal analysis, Data curation, Conceptualization.

## Declaration of competing interest

The authors declare the following financial interests/personal relationships which may be considered as potential competing interests:Biadge Kefale reports financial support was provided by 10.13039/501100004535Ethiopian Institute of Agricultural Research. Biadge Kefale reports a relationship with Ethiopian Institute of Agricultural Research that includes: employment. Biadge Kefale has patent pending to Grant number is not needed. Corresponding Author employed at Ethiopian Institute of Agricultural Research If there are other authors, they declare that they have no known competing financial interests or personal relationships that could have appeared to influence the work reported in this paper.
